# Deposition Rate Effect on Optical and Electrical Properties of Thermally Evaporated WO_3−x_/Ag/WO_3−x_ Multilayer Electrode for Transparent and Flexible Thin Film Heaters

**DOI:** 10.1038/s41598-020-65260-1

**Published:** 2020-05-20

**Authors:** Sang-Hwi Lim, Han-Ki Kim

**Affiliations:** School of Advanced Materials Science and Engineering, Sungkyunkwan University, 2066, Seobu-ro, Jangan-gu, Suwon-si, Gyeonggi-do 16419 Republic of Korea

**Keywords:** Materials science, Materials for devices

## Abstract

We investigated the deposition rate effect on the optical, electrical, and morphological characteristics of thermally evaporated WO_3−x_/Ag/WO_3−x_ (WAW) multilayer electrodes. By controlling the deposition rate of the WO_3−x_ and Ag layers, we can control the interface structure between WO_3−x_ and Ag and improve both the optical and electrical properties of the thermally evaporated WAW multilayer electrodes. At the optimized deposition rate of WO_3−x_ (2.5 Å/sec) and Ag (10 Å/sec), the symmetric WAW multilayer exhibited a high optical transmittance of 92.16% at a 550 nm wavelength and low sheet resistance of 3.78 Ω/square. During repeated bending, rolling, and twisting, there was no resistance change indicating the superior flexibility of WAW multilayer electrodes. As a promising application of the WAW multilayer electrodes, we suggested the transparent and flexible thin film heaters (TFHs) to substitute the high cost indium tin oxide-based TFHs. In comparison to the ITO-based TFHs, the WAW based TFHs showed higher convective heat transfer property and higher saturation temperatures are achieved at lower input voltages due to lower sheet resistance. This indicates that the WAW multilayer is suitable as the electrode for high performance transparent and flexible TFHs.

## Introduction

The importance of flexible and transparent conducting electrodes (TCEs) with high transmittance, low resistivity, good flexibility, and low materials cost has increased in the recently for high performance flexible and transparent displays, photovoltaics, and electronic devices^1–5^. Among the various applications of TCE, flexible and transparent thin film heaters (TFHs) have been considered to be one of the important applications. This is because the heating performances and reliability of flexible TFHs are critically dependent on the electrical, optical, and mechanical properties of TCE films^6–10^. Therefore, as a heating source for transparent windows, the importance of flexible and transparent TFHs attached to the window of automobiles and next-generation buildings covered with glass exterior has extensively increased. Typically, Sn-doped In_2_O_3_ (ITO) films fabricated by the magnetron sputtering process have been mainly employed as TCE for transparent TFHs^11,12^. Although ITO-based TFHs has been produced, the high cost of Indium element and brittleness of sputtered ITO film still remained as critical problems of ITO-based TFHs. To solve the problem of sputtered ITO electrode in TFHs, several types of TCE materials with low sheet resistance, high optical transmittance and superior mechanical flexibility have been intensively reported to replace ITO films. Metal-based TCE (metal mesh, metal nanowires and metal network), carbon-based TCE (carbon nanotube and graphene), polymer-based TCE, and hybrid TCE have been extensively reported as promising TCE materials to substitute the typical ITO electrode^2,13–16^. In particular, sputtered oxide-metal-oxide (OMO) multilayer electrodes, such as ITO/Ag/ITO, InZnSnO/Ag/InZnSnO, ZnO/Ag/ZnO, InZnO/Ag/InZnO, InSiO/Ag/InSiO, ITON/Ag-Ti/ITON, ITO/Ag/ZTO, CuO/Cu/CuO, and WO_3_/Ag/WO_3_ have been known to be important hybrid TCE^17–25^. In addition, the thermal evaporated SnO_2_/AgPdCu/SnO_2_, ATO/Ag/ATO, MoO_3_/Ag/MoO_3_, and WO_3_/Ag/WO_3_ multilayers also showed comparable electrical and optical properties to the sputtered OMO electrodes^20,26–28^. Regardless of deposition methods, the OMO multilayers have a high transmittance due to the suppression of surface plasmon resonance and destructive interference of reflective lights from interfaces and surfaces when the refractive index and thickness of dielectric oxide layers and reflective metal interlayer are adequate^29,30^. Moreover, due to the thin dielectric oxide layers and superior conductivity of the metal interlayer, the OMO multilayer could act as metal electrodes with a low resistivity. As a consequence, very thin OMO multilayers could be able to achieve high transmittance and low sheet resistance as well as good flexibility. For these reasons, the OMO multilayer electrode has been used as a possible substitute of conventional indium tin oxide (ITO) electrode for flexible optoelectronics applications^31–34^. However, the conventional sputtered OMO multilayer has serious problems such as degradation of the polymer-based flexible substrate and the soft organic layer beneath the OMO multilayer due to the bombardment of accelerated energetic ions, neutrals and radiative heat during the sputtering process^35–38^. Moreover, the high cost of indium based OMO multilayers can be a large obstacle for cost-effective production and fast diffusion of the indium element is problems for the reliability of devices. Therefore, the indium-free OMO multilayer TCE fabricated by using thermal evaporation is a more practical method to produce a low-damaged OMO multilayer. Although the feasibility of the thermally evaporated OMO electrode has been well reported, the effect of the deposition rate on electrical, optical, and morphological properties of the thermally evaporated OMO electrode was not investigated in detail^17–28,31–34^. In order to improve and control the electrical and optical properties of the thermally evaporated OMO electrode, an exact understanding of the deposition rate effect of each layer is imperative. However, the effect of the deposition rate on interface morphology, optical and electrical properties of the thermally evaporated OMO multilayer are rarely investigated compared to the effect of film thickness.

In this work, we investigated the effect of deposition rate on the electrical, optical and morphological properties of WO_3−x_/Ag/WO_3−x_ (WAW) multilayer deposited on a colorless polyimide (CPI) substrate during the thermal evaporation process. At a constant thickness of Ag (12 nm) and WO_3−x_ (30 nm) layers, we investigated the electrical, optical, and surface properties of thermal evaporated Ag single layer, WO_3−x_ single layer and WAW multilayer according to the deposition rate. In addition, we measured the resistance change of optimized WAW multilayer by using lab-designed several bending test systems to show good flexibility of the WAW multilayer electrodes. Furthermore, as a promising application of the WAW multilayer electrode, we suggested transparent and flexible TFHs which could be attached to glass windows. We investigated the correlation between performances of TFHs and electrical properties of the WAW multilayer electrodes to show the importance of thermally evaporated WAW electrode.

## Results

**Figure 1:**
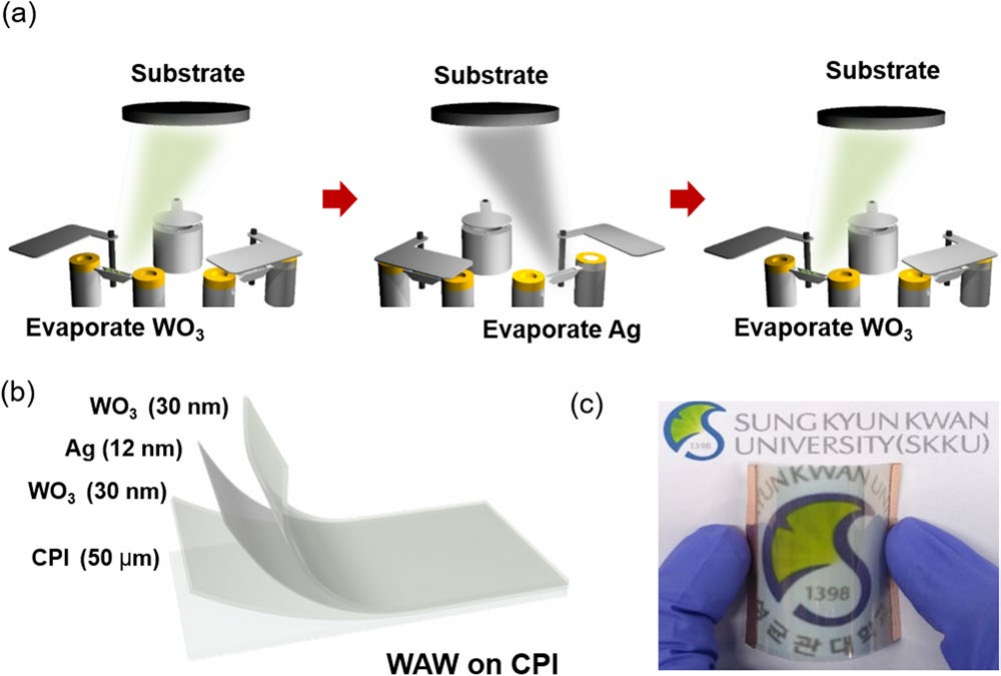
(**a**) Schematic illustration of the continuous thermal evaporation process to fabricate WAW multilayer films on flexible CPI substrate. By evaporation of WO_3−x_ and Ag sources and controlling shutters, symmetric WAW multilayers were fabricated. (**b**) Structure of WAW (30/12/30 nm) multilayer films on a 50 μm-thick CPI substrate. (**c**) The photograph shows a flexible and transparent TFH with an optimized WAW electrode. (**a**) and (**b**) Were drawn by using a Rhino 6, which is 6^th^ version of this drawing program and URL link is http://www.rhino3d.com.

Figure 1a schematically illustrates the continuous thermal evaporation process to fabricate the WAW multilayer in a multi-boat thermal evaporation system using WO_3_ and Ag sources. In the continuous thermal evaporation process, the bottom WO_3−x_ layer, Ag interlayer, and top WO_3−x_ layer were deposited on a 50 μm-thick commercial CPI substrate (Kolon industry, LTD) under base pressure of 1.0 × 10^−6^ Torr by controlling the shutter and power of tungsten boats. Detailed evaporation conditions to prepare the WAW multilayer were summarized in Table 1. As illustrated in Fig. 1b, the WAW multilayer had a symmetric structure that was desirable for the antireflection effect in high transmittance^39^. Fig. 1c is a photograph of the flexible and transparent TFH fabricated on the flexible WAW (30/12/30 nm) electrode prepared at an optimal deposition rate. Due to the high transmittance and good flexibilities, the symbol of Sungkyunkwan university is clearly seen through a bent WAW coated CPI substrate. Before the optimization of the WAW multilayer, we investigated the optical and electrical properties of each WO_3−x_ and Ag layers, respectively, according to the deposition rate. Among several parameters in the optimization of the WAW multilayer, the film thickness and interface morphology are key parameters affecting the optical, electrical, and mechanical properties of the WAW multilayer^25,39,40^. Hong *et al*., reported that the optimized thickness of WO_3−x_ and Ag layers in WAW multilayer is 30 nm and 12 nm, by using finite-difference time-domain (FDTD) simulation and experiments^39^. However, the effect of evaporation speed and interface morphology of optical and electrical properties of the WAW multilayer are rarely investigated compared to the effect of the film thickness. Therefore, in this experiment, we fixed the thickness of the WO_3−x_ and Ag layers as 30 nm and 12 nm and focused on the effects of interface morphology on the performance of the WAW multilayer by controlling the evaporation speed of the WO_3−x_ layer and Ag interlayer. Fig. S1a (Supporting information) shows the optical transmittance of 12 nm thick Ag layer as a function of the deposition rate from 0.25 to 1.0 nm/sec at a wavelength range of 400 to 800 nm. Ag single films were semi-transparent due to the thinner film thickness (12 nm) than the optical skin depth of Ag (~20 nm)^41^. With increasing deposition rate of the Ag layer from 0.25 to 1.0 nm/sec, the optical transmittance at a wavelength of 550 nm slightly increased from 43.57% to 48.96% due to the decrease of light scattering on the surface of the smooth Ag layer. At a low deposition rate, the randomly disconnected Ag islands led to light scattering at the rough surface. Fig. S1b,c are the Hall measurement results obtained from the 12 nm thick Ag singly layer with increasing the deposition rate. With increasing the deposition rate from 0.25 to 1.0 nm/sec, the sheet resistance and resistivity of the Ag single layer gradually decreased. The decreased sheet resistance and resistivity of the Ag single layer grown at a higher deposition could be attributed to the increased carrier mobility as shown in Fig. S1c. However, the carrier concentration of Ag thin films was almost identical regardless of the deposition rate. At a high deposition rate, the evaporated Ag atoms were uniformly dispersed on the surface and formed a well-connected and smooth Ag layer. Therefore, a faster deposition rate led to the formation of a more smooth Ag layer with larger grain and better connectivity. As a consequence, an Ag thin film grown at a deposition rate of 1.0 nm/sec showed the lowest sheet resistance of 3.76 Ohm/square and resistivity of 4.51 × 10^−6^ Ohm-cm. Based on transmittance (T) at a wavelength of 550 nm and sheet resistance (R_sh_) of the Ag single layer, the figure of merit (FOM = T^10^/R_sh_) values were calculated according to deposition rate^42^. As shown in Fig. S1d, the Ag single layer grown at a deposition rate of 1.0 nm/s had the highest FOM value. Therefore, we determined that the optimal deposition rate of the thermally evaporated Ag single layer was 1.0 nm/sec to obtain high-performance WAW multilayer electrodes. Fig. S2a shows the optical transmittance at a wavelength between 400 and 800 nm of the 30 nm-thick WO_3−x_ film on a glass substrate according to the WO_3−x_ deposition rate from 0.25 to 1.0 nm/sec. The WO_3−x_ x layer had the highest optical transmittance at a deposition rate of 0.25 nm/sec, although the transmittance differences according to the deposition rate were small. Figure S2b,c also shows the Hall measurement results obtained from 30 nm thick WO_3−x_ thin films evaporated on a glass substrate with increasing evaporation speed. As the deposition rate of WO_3−x_ increased, the mobility of the WO_3−x_ single layer increased while the carrier concentration of the WO_3−x_ film was almost identical. Therefore, a decrease of sheet resistance and resistivity of the WO_3−x_ single layer could be attributed to increased mobility. Like the Ag films, the WO_3−x_ film tends to grow in a dispersed manner rather than agglomerated and WO_3−x_ film. As a consequence, the evaporated WO_3−x_ film grown at a deposition rate of 1.0 nm/s shows the lowest sheet resistance of 670 Ohm/square and resistivity of 2.01 × 10^−3^ Ohm-cm. Furthermore, based on the R_sh_ and T at a wavelength of 550 nm for the WO_3−x_ layer, the FOM values were calculated. As shown in Fig. S2d, the WO_3−x_ single layer with a deposition rate of 1.0 nm/s had the highest FOM value due to the low sheet resistance and high optical transmittance. However, because of the sheet resistances of the OMO multilayer were determined by the metal layer^29^, optical properties are much more important factors as the oxide layer of the OMO multilayer. Therefore, we determined the optimal deposition rate of the thermally evaporated WO_3−x_ layer is 0.25 nm/sec to obtain high optical transmittance of WAW multilayer electrodes.

**Table 1:** Detailed thermal evaporation conditions to coat WAW multilayer on the CPI substrates.

Material	Thickness [nm]	Density [g/cm3]	Z-factor	Tool factor [%]	Base pressure [torr]	RPM	Deposition rate [nm/sec]
WO_3−x_	30	7.16	*1.00	159	<1.0 × 10^−6^	11	0.25
Ag	12	10.5	0.529	151	<1.0 × 10^−6^	11	Variable
WO_3−x_	30	7.16	*1.00	159	<1.0 × 10^−6^	11	0.25

The bottom WO_3−x_ layer, Ag interlayer, and top WO_3−x_ layer were consecutively evaporated on a CPI substrate by using an identical evaporation system at room temperature without breaking the vacuum.

**Figure 2:**
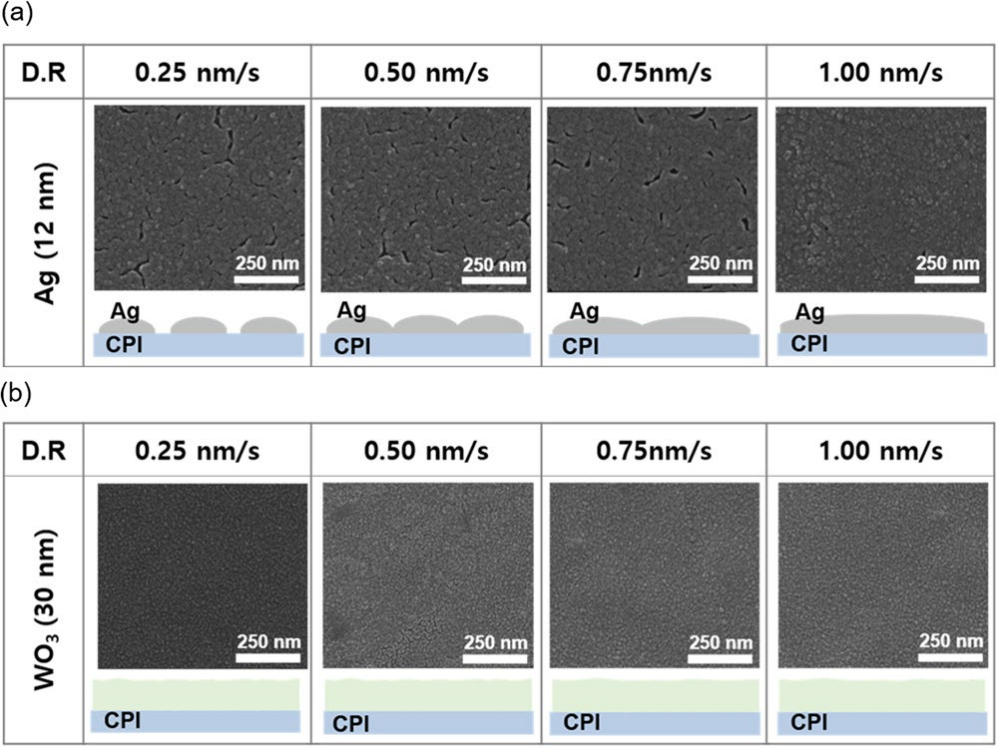
Surface FE-SEM images of (**a**) Ag and (**b**) WO_3−x_ films with increasing deposition rate from 0.25 nm/s to 1.0 nm/s. The bottom images indicate a schematic cross-sectional structure of the evaporated Ag and WO_3−x_ films grown at different deposition rates.

Figure 2a,b shows the surface FE-SEM images and schematically illustrates a cross-sectional view of the evaporated Ag and WO_3−x_ films at room temperature with increasing deposition rate, respectively. As the deposition rate of the Ag increased from 0.25 nm/s to 1.0 nm/s, the grain size also slightly increased and formed a smoother surface. Since the grain size of the Ag layer increased, voids or uncoated areas between islands decreased and the connectivity of the islands increased. Therefore, a high deposition rate of the Ag layer improved the electrical and optical properties. In the case of the WO_3−x_ film, the relationship between deposition rate and surface roughness was hard to find in the surface FE-SEM images due to a very small grain size. Even at different deposition rates, all WO_3−x_ films showed similar amorphous like surface morphology. Therefore to achieve a smooth interface with good quality, the high Ag deposition rate is needed when depositing the Ag interlayer.

**Figure 3:**
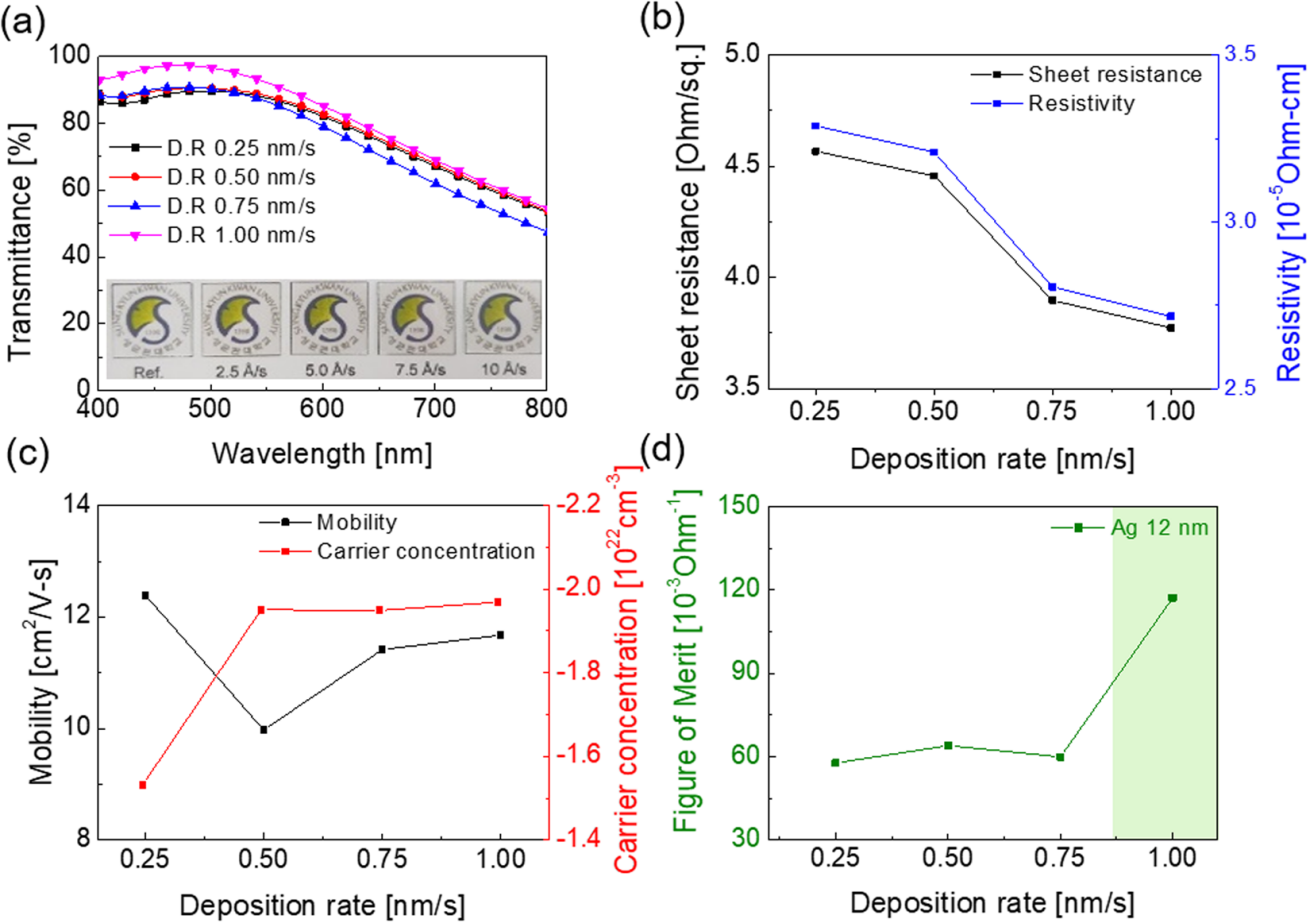
(**a**) Sheet resistance, resistivity, (**b**) mobility and carrier concentration of thermally evaporated WAW multilayer films on a glass substrate with increasing Ag deposition rate from 0.25 to 1.0 nm/sec at a constant WO_3−x_ deposition rate of 0.25 nm/sec. (**c**) Optical transmittance, and (**d**) FOM values calculated from sheet resistance (Rsh) and optical transmittance (T) of the WAW multilayer films.

Figure 3a also shows the optical transmittance of the thermally evaporated WAW multilayer electrodes with increasing deposition rate of the Ag interlayer. Due to the anti-reflection effect generated in the symmetric DMD structure, the thermal evaporated WAW multilayer films demonstrated a high transmittance in visible wavelength region. The dielectric WO_3−x_ layer had higher relative permittivities than the Ag interlayer, it can suppress surface plasmons coupling at the interface of the metal and dielectric, and consequently, increased the optical transmittance of the WAW multilayer film^29^. Furthermore, the destructive interference between the reflected lights from the interfaces had a large influence on the transmittance of the DMD structure^43^. For the destructive interference, flat and well-aligned interfaces that light reflected is crucial. Therefore, able to deducing that decreasing the roughness of interface layers between the top WO_3−x_ layer and Ag interlayer is key to the achievement of high optical transmittance of WAW multilayer film, and as noted previously, these can be achieved by increasing the deposition rate of the Ag interlayer. The transmittance data of the WAW multilayer had good accordance with this expectation. The WAW multilayer film with the Ag deposition rate of 0.25 nm/s showed the lowest optical transmittance of 87.52% at a wavelength of 550 nm due to a rough interface. However, when the deposition rate of Ag was 1.00 nm/s, it exhibited the highest optical transmittance of 92.16% at a wavelength of 550 nm due to the smooth interface. Figure 3b,c show the Hall measurement results of the WAW multilayer films prepared with a function of the deposition rate of the Ag interlayer at a fixed deposition rate (0.25 nm/sec) of the top and bottom WO_3−x_ layers. As summarized in Table 2, when we evaporated the Ag interlayer at a deposition rate of 0.25 nm/s, the WAW multilayer electrode showed the highest sheet resistance of 4.566 Ohm/square and resistivity of 3.288 × 10^−5^ Ohm·cm. When the evaporation speed of the Ag interlayer increased, both sheet resistance and resistivity of the WAW multilayer electrode decreased. At the deposition rate of 1.0 nm/sec, the WAW multilayer showed the lowest sheet resistance of 3.773 Ohm/square and resistivity to 2.716 × 10^−5^ Ohm·cm. Compared to the previously reported WAW multilayer electrode, this WAW multilayer showed a lower sheet resistance and resistivity^39,40,44^. In addition, the optimized WAW electrode showed better electrical properties than the amorphous ITO films even though both films were prepared at room temperature (Table 2). Based on the sheet resistance and optical transmittance at a wavelength of 550 nm, the FOM values were calculated according to the deposition rate of the Ag interlayer. As shown in Fig. 3d, with the increase of the Ag deposition rate from 0.25 nm/s to 1.0 nm/s, the WAW multilayer film exhibited the increased FOM value from 57.75 × 10^−3^ Ohm^−1^ to 117.2 × 10^−3^ Ohm^−1^. Moreover, compared to previously reported OMO multilayer electrodes (Table 3), the optimized WAW multilayer electrode has better optoelectrical properties. Specifically, the WAW multilayer film prepared at a low Ag deposition rate (0.25 nm/s) shows similar FOM value to previously reported OMO multilayers (41.4–70.7 × 10^−3^ Ohm^−1^) in Table 3. However, after optimizing the Ag deposition rate (1.0 nm/s), the WAW electrode exhibit the highest FOM value among multilayer TCEs due to high optical transmittance of 92.16% at a wavelength of 550 nm and low sheet resistance of 3.773 Ohm/square. These superior optoelectrical properties of optimized WAW multilayer film originated from the enhancement of Ag islands connectivity and interface flatness that affects both electrical conductivity and optical transparency by a high Ag deposition rate of 1.0 nm/s. As a result, unlike the conventional trade-off method that inevitably sacrificed one property to enhance one of the optical or electrical properties by modulating the thickness of the metal layer, we were able to enhance both the optical and electrical properties at the same time by increasing the Ag deposition rate.

**Table 2:** The electrical and optical properties of the thermally evaporated WAW multilayer films on a glass substrate with increasing Ag deposition rate from 0.25 nm/s to 1.0 nm/s with a constant WO_3−x_ deposition rate of 0.25 nm/s.

WAW Ag deposition rate	0.25 nm/s	0.50 nm/s	0.75 nm/s	1.00 nm/s	ITO (100 nm)
Sheet resistance [Ohm/sq.]	4.566	4.458	3.896	3.773	47.4
Resistivity [10^−5^ Ohm-cm]	3.288	3.210	2.805	2.716	47.4
Mobility [cm^2^/V^−s^]	12.39	10.02	11.42	11.68	11.5
Carrier concentration [10^22^ cm^−3^]	1.532	1.939	1.949	1.969	0.115
550 nm wavelength	87.52	88.21	86.46	92.16	76.21
400~800 nm average	77.38	78.27	75.16	81.60	76.42
Figure of Merit [10^−3^ Ohm^−1^]	57.75	64.00	59.92	117.2	1.394

FOM values calculated using the sheet resistance (R_sh_) and optical transmittance (T) of the WAW multilayer films.

**Table 3:** Structure, sheet resistance, transmittance and the figure of merit values of the WAW and reported multi-layer TCEs.

OMO Structure	Sheet resistance (Ohm/square)	Transmittance (%, at 550 nm)	Figure of merits (10^−3^ × T^10^/R_sh_)	Ref.
WO_3−x_/Ag/WO_3−x_	3.77	92.16	117	This work
ITO/Ag/ITO	4.4	86.54	49	^17^
IZTO/Ag/IZTO	4.99	86	44	^18^
IZO/Ag/IZO	4.15	87	60	^20^
ISO/Ag/ISO	5.26	84.8	41.4	^21^
SnO_2_/APC/SnO_2_	9.42	91.14	42.0	^26^
ATO/Ag-Ti/ATO	6.91	90.24	51.8	^27^
ZTO/APC/ZTO	3.43	80.99	35.4	^31^
WO_3_/Ag/WO_3_	7.22	93.5^*^ (at 510 nm)	70.7	^39^

**Figure 4:**
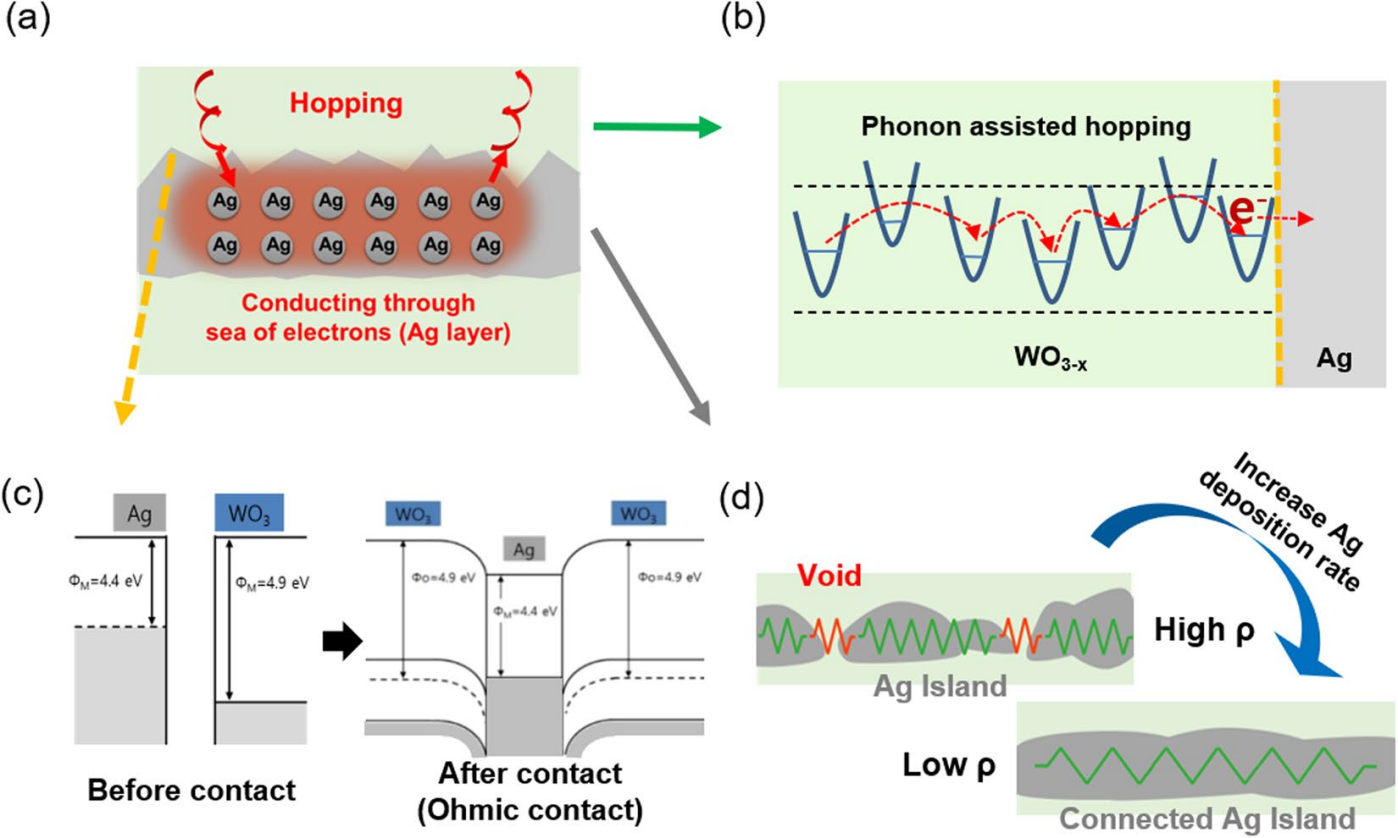
(**a**) Conduction mechanism of the WAW multilayer films. (**b**) Conduction mechanism of the WO_3−x_ layer is phonon-assisted hopping and the WO_3−x_ layer is thin enough for the electron to pass through with negligible voltage drop. (**c**) Ohmic contact a WO_3−x_/Ag interface. (**d**) Charge conduction properties in the Ag layer is superior due to the well-connected island structure and charge carriers were free to move through the sea of electrons.

Figure 4a is a schematical illustration of the conduction mechanism of the WAW multilayer film. The current flow in the WAW multilayer film could be explained as described below. The first current path was the top WO_3−x_ layer as illustrated in Fig. 4b. The WO_3−x_ film was a well-known n-type semiconductor conducted by hopping of localized electrons from one metal ion to another, which was in different valence states^45–47^. The resistivity of the 30 nm thick WO_3−x_ layer had a resistivity of 9.574 × 10^−2^ Ohm-cm as a deposition rate of 0.25 nm/sec. Therefore, the electron was conducted through the thin WO_3−x_ layer without a potential drop due to negligible resistance of vertical conduction, and reaching WO_3−x_ /Ag interface. Due to the work function of the top WO_3−x_ layer and Ag interlayer as shown in Fig. 4c, the Ohmic contact formed at the junction of the WO_3−x_ and Ag interlayer. Therefore, an interface of these two layers followed Ohm’s law and have a linear current-voltage relationship. As a result, electrons can flow easily in both directions at the interface of the WO_3−x_ layer/Ag interlayer. When electrons were injected into the Ag interlayer through this process, electrons were free to move through the Ag interlayer. Therefore, the Ag interlayer performs as a lateral charge conducting layer of the WAW multilayer electrode and this is the main reason why the WAW multilayer film has superior electrical properties as the Ag metal film. Moreover, as mentioned above, the WAW multilayer film that was fabricated at slow evaporation has more rough interfaces compared to the Ag interlayer prepared at a faster deposition rate due to the more number of disconnected Ag islands and voids. The electrical conductivity of the WAW multilayer was not only obstructed by voids that act like high resistance resistor (Fig. 4d), but also deteriorated by the scattering of electrons at the interface, surface, and grain boundary induced by rough morphology^48^. From this perspective, because the WO_3−x_ surface roughness change is relatively small considering the Ag surface roughness change, the deposition rate of the WO_3−x_ layer could not affect on the WAW multilayer electrode. Therefore, to optimize the performance of the WAW multilayer, it is important to enhance the Ag electrical properties and reduce surface roughness than enhance the WO_3−x_ electrical properties. Furthermore, we measured the work function of the optimized WAW multilayer film using the Kelvin probe force microscopy (KPFM). The optimized WAW electrode has a work function of 4.49 eV (Fig. S3b) and similar to the work function of the conventional of FTO and ITO electrode (4.2–4.7 eV)^49^. Moreover, considering conduction band minimum energy of general ETLs such as ZnO (4.4 eV)^50^, SnO_2_ (4.5 eV)^51^, and PCBM (4.3 eV) for the organic or perovskite photovoltaics^49^, it will be suitable as transparent cathode.

**Figure 5:**
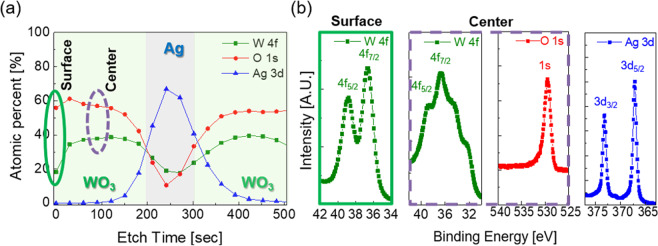
(**a**) XPS depth profile of the thermally evaporated WO_3−x_ (30 nm)/Ag (12 nm)/WO_3−x_ (30 nm) multilayer film on a CPI substrate. XPS core-level spectra of (**b**) WO_3−x_ 4 f (surface of WO_3−x_ layer), WO_3−x_ 4 f (inside of WO_3−x_ layer), O1s (inside of WO_3−x_ layer), and Ag 3d.

Figure 5a is the X-ray photoelectron spectroscopy (XPS) depth profile of the WO_3−x_ (30 nm)/Ag (12 nm)/WO_3−x_ (30 nm) multilayer film prepared at optimized thermal evaporation conditions. In the XPS depth profile analysis, constant tungsten and oxygen weight percentages in both the bottom and top WO_3−x_ layers was observed. In addition, the symmetric composition of W and O at both top and bottom WO_3−x_ layers indicates that the thickness and process condition of both WO_3−x_ layers were controlled identically during the thermal evaporation process and corresponds with the cross-sectional FE-SEM analysis of the WAW multilayer film (Fig. S4). In this profile, the weight percentages of W and O at the surface of the top WO_3−x_ layers and inside of the top WO_3−x_ layers were different. The relative composition of the W/O was approximately 1/3 obtained from the surface of the top WO_3−x_ layer, while the inside composition of the WO_3−x_ layer was higher than 1/2. Figure 5b shows the measured core-level spectra of the W 4 *f* at the surface of the top WO_3−x_ layer and inside of the top WO_3−x_ layer. At the surface of the top WO_3−x_ layer, W 4 *f* binding energies were located at 33.38 eV (W 4*f*_7/2_) and 35.51 eV (W 4*f*_5/2_) and showed similar intensity to the previously reported WO_3_ thin film^52^. However, inside of the top WO_3−x_ layer, the W4*f* binding energies were located at 36.75 eV (W 4*f*_7/2_) and 38.3 eV (W 4*f*_5/2_). The intensity of the W*f* spectra was similar to the intensity pattern of the WO_2_ thin film, which was also reported by O. Y. Khyzhun^52^. These phenomena happened because of the vacuum pressure difference of tungsten and oxygen during thermal evaporation. In the thermal evaporation process, the partial pressure of O was higher than W and this made the O atom hard to evaporate from the WO_3_ source compared to the W atom. Therefore, the whole WO_3−x_ layer has a similar stoichiometry of WO_2_. However, the top WO_3−x_ layer maintained stoichiometry of the WO_3−x_ because of oxidation after exposure of the top layer in ambient. In addition, the binding energy of the Ag located at 373.18 eV (3*d*_3/2_) and 368.43 eV (3*d*_5/2_) showed a typical characteristic of the metallic Ag layer^53^. This result indicates that the Ag interlayer was well fabricated as a metallic layer without interfacial reactions with the WO_3−x_ layer.

**Figure 6:**
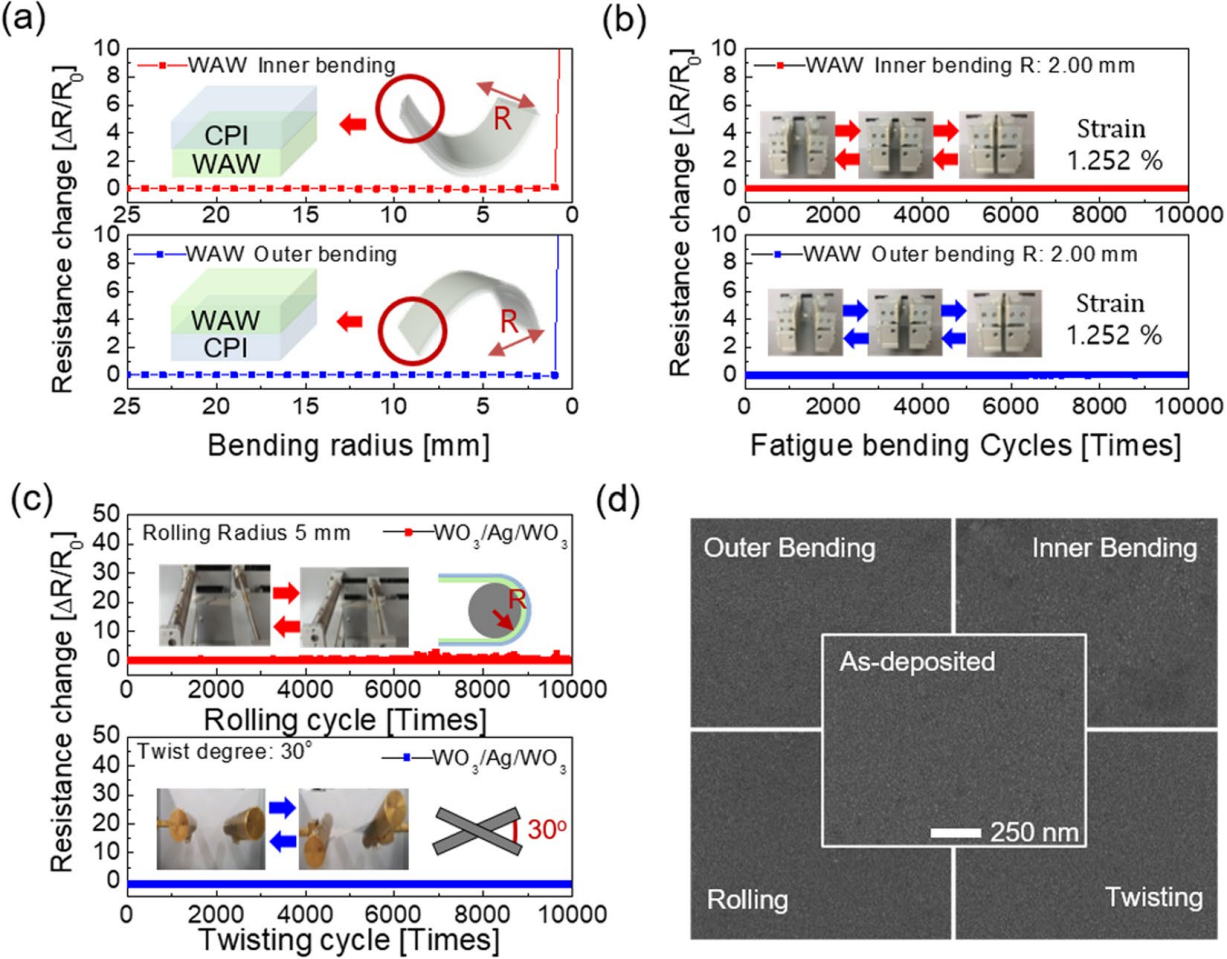
(**a**) Resistance changes during outer/inner bending tests of the WAW multilayer film coated on CPI substrate with decreasing outer/inner bending radius. The inset showed the WAW sample experiencing compressive (inner) and tensile (outer) strains. (**b**) Repeated inner and outer bending tests of the WAW sample as a function of bending cycles. The inset picture showed the bending steps during repeated bending tests. The bending radius of both the outer and inner bending fatigue tests was kept at 2 mm. (**c**) Resistance changes of rolling (top) and twisting (bottom) fatigue tests of the WAW multilayer film with increasing bending cycles. Rolling radius and a twist angle were kept at 5 mm 30°. The inset picture showed the rolling and twisting test steps. (**d**) Surface FE-SEM images of as-deposited WAW multilayer film (center) and after various bending tests. (**a**) Was drawn by using a Rhino 6, which is 6^th^ version of this drawing program and URL link is http://www.rhino3d.com.

To demonstrate the outstanding flexibility and durability of the WAW multilayer coated on CPI substrate, we carried out mechanical tests using lab-made inner/outer bending, rolling and twisting test systems **(**Fig. 6). All the bending test systems could *in-situ* measure resistance change (ΔR) of the WAW samples when the samples experienced the bending, rolling and twisting tests because both ends of samples were tightly clamped in the electrodes. Figure 6a shows the resistance changes of the WAW multilayer as a function of the bending radius during the outer and inner bending test (Inset of Fig. 6a). In this measurement, the resistance change (ΔR) of the measured samples are expressed as (R-R_0_), where R_0_ and R indicate the initially measured resistance and measured resistance after the bent of the sample, respectively. The WAW multilayer showed a constant resistance change even at the bending radius of 2.0 mm. However, further decrease of bending radius led to an abrupt increase of resistance change indicating the formation of crack and physical separation or delamination of samples from the substrate. The peak strains applied to the WAW multilayer could be calculated by using the following equation^31^.

1 $${\rm{Strain}}=\frac{{d}_{WAW}+{d}_{CPI}}{2{\rm{R}}}\times 100 \% $$

where, R is the bending radius, *d*_WAW_ and *d*_CPI_ are the thickness of the WAW multilayer (72 nm) and the CPI substrate (50 μm), respectively. Both inner and outer bending with a 2.0 mm radius resulted in a 1.252% peak strain. The repeated inner and outer fatigue bending tests were carried out at a constant outer and inner bending radius of 2 mm as shown in Fig. 6b. There was no resistance change in the WAW multilayer regardless of the bending mode due to the superior flexibility of the WAW multilayer. Furthermore, the rolling and twisting fatigue tests were carried out with a rolling radius of 5 mm and a twisting angle of 30° to show the stable operation of the WAW multilayer even at severe bending conditions (Fig. 6c). Even after repeated 10,000 times rolling and twisting tests, there was no resistance change due to the good flexibility of the WAW multilayer. Figure 6d exhibits the surface FE-SEM images of the WAW multilayer before and after repeated bending tests for 10,000 times. Despite various repeated fatigue tests, the WAW multilayer showed identical surface morphology of the as-deposited sample (center) without any surface defects, such as cracks, delamination or protrusions. Based on the bending test results, we found that the thermally evaporated WAW multilayer played an important role as the flexible electrodes in the highly flexible TFHs.evaporated WAW multilayer played an important role as flexible electrodes in highly flexible TFHs.

**Figure 7:**
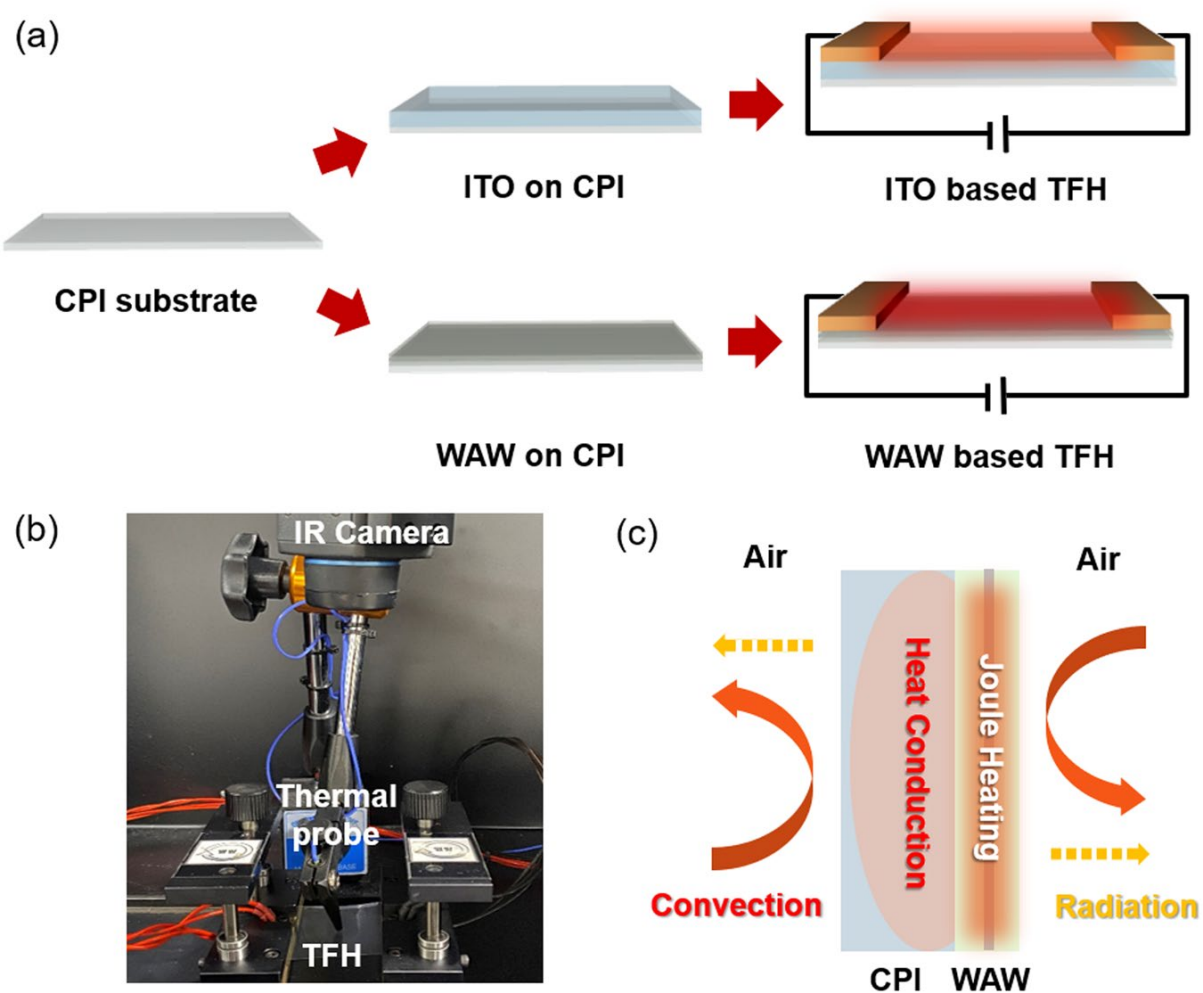
(**a**) Fabrication process of the WAW multilayer-based TFH and ITO-based TFH (reference). (**b**) Thermocouple and IR camera were used to monitor the temperature of the WAW multilayer-based TFH and ITO-based reference sample. (**c**) Heating mechanism of WAW-based TFHs. (**a**) Was drawn by using a Rhino 6, which is 6^th^ version of this drawing program and URL link is http://www.rhino3d.com.

To investigate the viability of the WAW multilayer film as a flexible and transparent electrode for the TFHs, we fabricated the typical TFHs with sizes of 25 × 25 mm^2^ as shown in Fig. 7a. To apply the current and DC voltage into the WAW electrode efficiently, we attached Cu electrodes on both ITO- and WAW-based TFHs. The temperature profile of the TFHs was monitored by a thermocouple mounted on the surface of the TFHs and infrared (IR) thermal images (Fig. 7b). As illustrated in Fig. 7c, when power is applied to the WAW multilayer-based TFHs, heat is generated by Joule heating and transferred to the CPI substrate by conduction. At the same time, small amounts of heat were released by convection and radiation from both surfaces of TFHs. Eventually, the quantity of generated heat (input power) and released heat became equal and reached an equilibrium temperature, where the temperature of TFHs was saturated.

**Figure 8:**
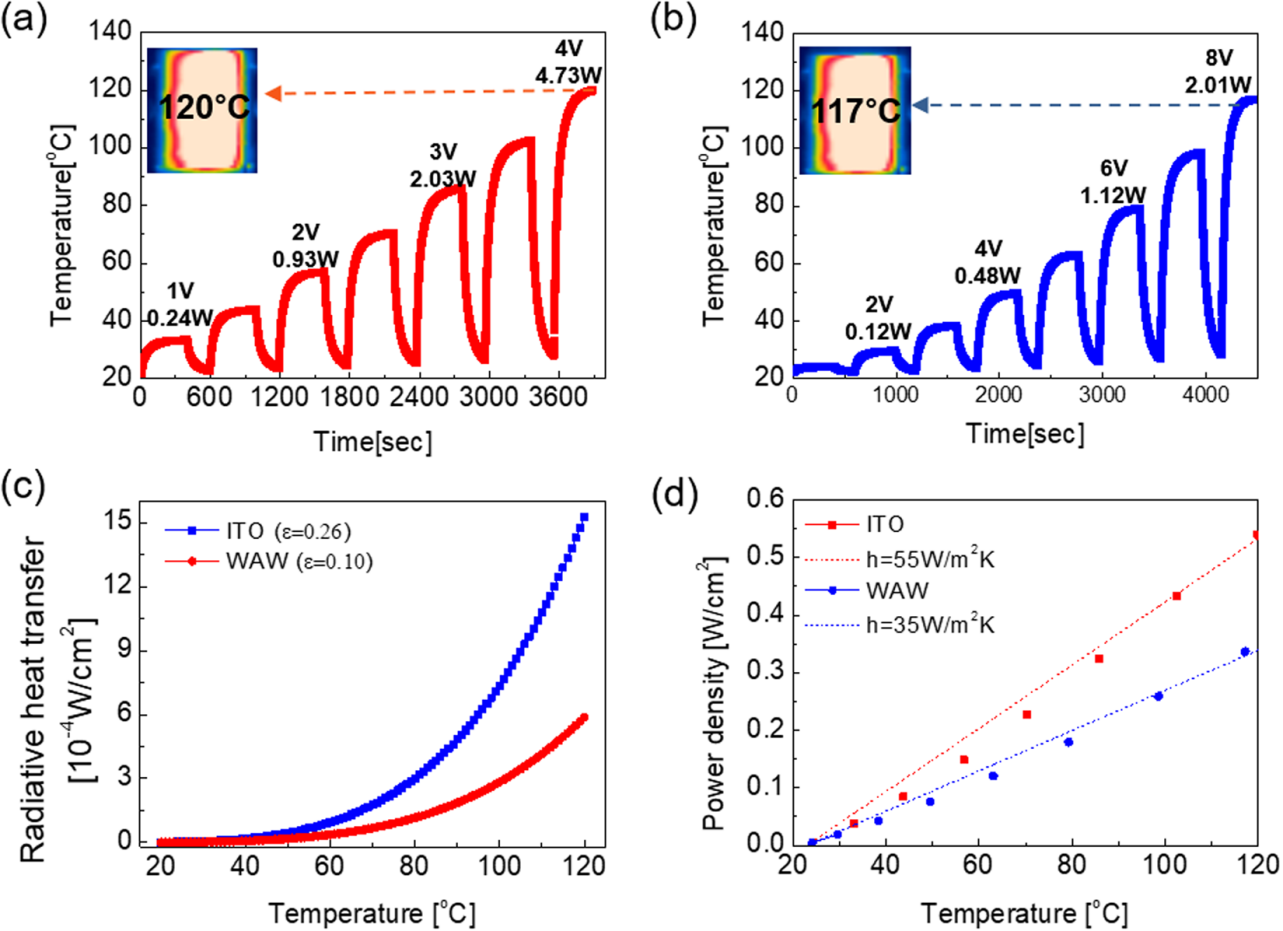
(**a**) Temperature profiles of the WAW-based TFHs and (**b**) amorphous ITO-based TFHs according to the time. (**c**) Radiative heat dissipation density of WAW-based TFHs and amorphous ITO-based TFHs as a function of saturated temperature. (**d**) The power density of WAW-based TFHs and amorphous ITO-based TFHs as a function of saturated temperature.

In order to assess the efficiency of WAW-based TFH, we compared the heating-cooling profile of the WAW-based TFHs and ITO-based TFHs as shown in Fig. 8. When 1 V DC voltage was supplied to the WAW-based TFHs, the temperature gradually increased and saturated at 24.2 °C. With increasing input voltage from 1 V to 4 V, the saturation temperature of WAW-based TFHs increased from 24.2 °C to 120 °C. Compared to amorphous ITO-based TFHs, the WAW-based TFHs achieve 120 °C with a low DC voltage due to low sheet resistance^32,54^. In order to analyze the heating properties of TFHs in detail, we investigated the relationship between heating efficiency and saturation temperature when the input power was identical. All types of heater, which included TFHs transformed all input power as energy in the form of heat as described by Bae *et al*., Described. The balance between the input power that was transformed into generated heat, and released heat can be expressed as below^55^.

2 $$mc\frac{{\rm{d}}T(t)}{{\rm{d}}t}=P-({Q}_{c}+{Q}_{r})$$

In Eq. (2), *m, c, T* and *t* are the mass of TFHs, specific heat capacity, the temperature of the TFHs and time, respectively. P*, Q*_*c*_, an*d Q*_*r*,_ are the input power, which is voltage multiplied with current, the convective power and radiative power losses, respectively. Here, the convective heat power loss (Q_c_) is expressed by,

3 $${Q}_{c}={h}_{c}A(T-{T}_{i})={h}_{c}A\Delta T$$

Where ${h}_{c}$ is the convective heat-transfer coefficient, A is the surface area, and T_i_ is the initial surface temperature, which is identical with room temperature^55^. Considering non-blackbody, the emitted radiant energy loss $({Q}_{r})$ from the surface of TFHs could be expressed by,

4 $${Q}_{r}=\varepsilon \sigma A({T}^{4}-{T}_{i}^{4})={h}_{r}A(T-{T}_{i})$$

where ε is the surface emissivity, σ the Stefan-Boltzmann constant (5.67 × 10^−8^ W-m^−2^-C^− 4^), and ${h}_{r}$ is the radiative heat transfer coefficient^55^. As Koubli *et al*.^56^. and K. Sun *et al*.^57^. reported, the WAW multilayer and ITO film without heat treatment have low surface emissivities of 0.09~0.10 and 0.21~0.26, respectively. By using those values, we calculated the amount of heat dissipation by radiation from TFHs (Fig. 8c). As shown in Fig. 8c,d, the calculated heat dissipation by radiation was negligible for the rough calculation because it had a 2~3 smaller order of magnitude compared to the total heat dissipation (Fig. 8d). Therefore, it is acceptable that the presumed total heat dissipation from TFH is the same with heat dissipation by convection as (Q_r_ = 0). When enough time passed, the temperature of TFHs had been saturated ($T={T}_{s}$) by the balance between the input power and heat power loss ($dT(t)/d{t}$ = 0). Therefore, we can express Eq. 2 like shown below, using Eqs. 3 and 4 and conditions mentioned above,

5 $$P/A=Q/A={h}_{c}({T}_{s}-{T}_{i})$$

6 $$\frac{P}{A({T}_{s}-{T}_{i})}={h}_{c}$$

**Figure 9:**
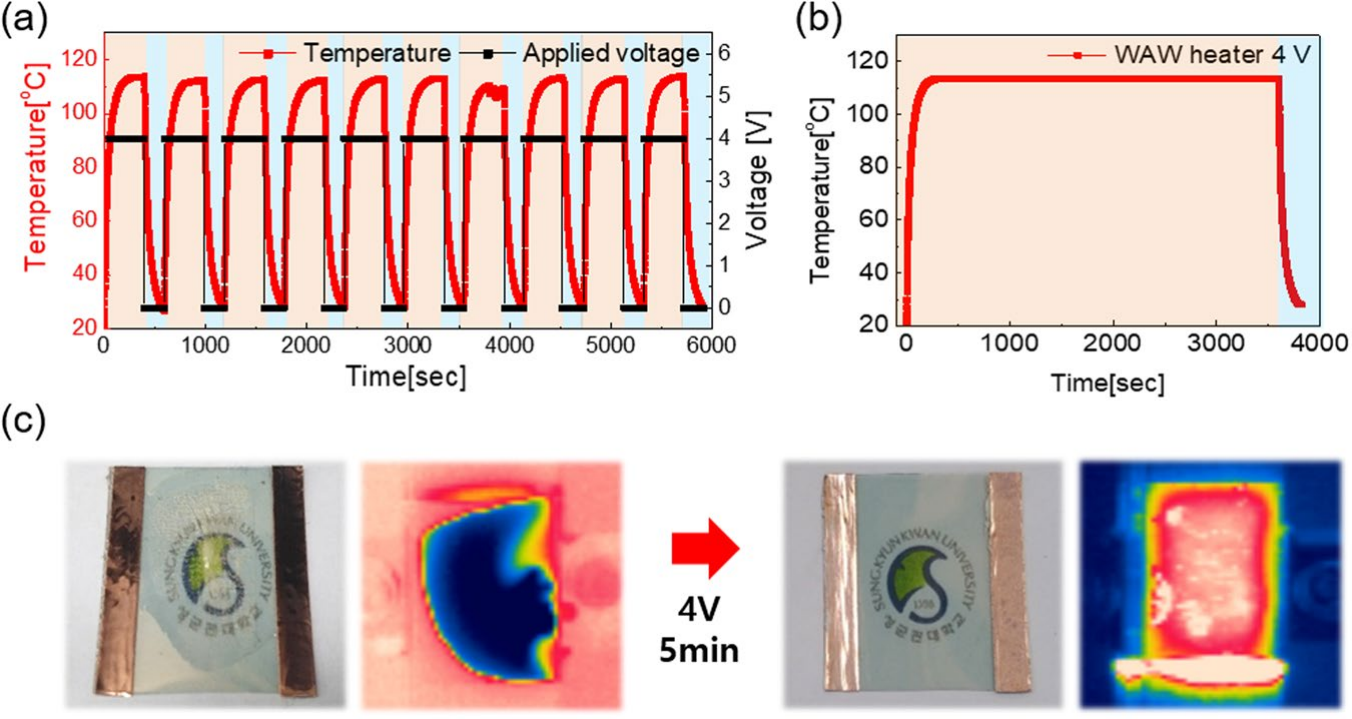
(**a**) The WAW-based TFHs showed the same heating-cooling temperature profiles during 10 repeated cycles. (**b**) show the durability of WAW-based TFHs and the ability to maintain saturation temperatures during 1-hour heating. (**c**) Photograph and IR image of before and after the deicing test.

In Eq. (6), when we assumed that input power ($P$), initial temperature (${T}_{i}$), and the dimension ($A$) of the TFHs are identical with other comparisons TFHs, one with lower saturation temperature has higher convective heat transfer coefficient (${h}_{c}$) than others. Furthermore, Eq. (5) shows us when we assumed that saturation temperature (${T}_{s}$), initial temperature (${T}_{i}$), and dimension ($A$) of the TFHs are same with other comparison targets, the input power of TFHs with higher convective heat coefficient (${h}_{c}$) is higher. In other terms, TFHs with higher convective heat transfer coefficient emit heat more at the same operation temperature (saturation temperature). As shown in Fig. 8d, the convective heat transfer coefficient of WAW-based TFHs is higher than 1.5 times of ITO-based TFHs. Compared to previously reported TCE-based TFHs (Table S1), the WAW-based TFHs achieved a saturation temperature over 100 °C at a relatively low DC voltage and have higher convective heat transfer coefficient. Consequently, WAW-based TFHs are more effective and safe heater than a-ITO and other TCEs based TFHs because it emits the same amount of heat at relatively lower operation temperature without increasing temperature more which help to prevent fire or short-circuit of the heater. In order to demonstrate the stability of WAW-based TFHs, consecutive 10 cycles of heating and cooling tests and a one-hour heating test were conducted as shown in Fig. 9a. The saturation temperature and heating-cooling profiles of the WAW-based TFH were maintained during the cycles of repeated application and removal of DC 4 V. Figure 9b shows the durability of the WAW-based TFHs and the ability to maintain saturation temperature during one-hour heating. These temperature profiles demonstrate that the WAW multilayer is a good replacement for the conventional a-ITO TCE. Besides, we conducted a defrosting test of the WAW-based TFHs and confirmed by photographs and IR images (Fig. 9c). WAW-based TFHs was stored in a refrigerator for 120 minutes for frost formation. When DC 4 V was applied for 5 minutes, the frost on the surface of the WAW based TFHs was melted and evaporated completely.

## Conclusion

In conclusion, we investigated the feasibility of thermally evaporated WAW multilayer electrode for application as flexible TCE without indium for high-performance flexible TFHs and can be used as TCE for the next-generation optoelectronics and photovoltaics. The optical and electrical properties of the top and bottom WO_3−x_ layers and Ag interlayer according to the deposition rate were investigated prior to the optimization of the deposition rate of each layer in the WAW multilayer TCE. Based on the optical, electrical, morphological and structural properties of WAW multilayer electrodes, we achieved an optimized WAW multilayer with high optical transmittance of 92.16% at the wavelength of 550 nm and low sheet resistance of 3.77 Ohm/square. Furthermore, we showed these superb properties were due to a smooth surface and interface morphology affected by a deposition rate of Ag interlayer, as well as symmetric structure. In addition, the thermally evaporated WAW multilayer showed superb mechanical flexibility in various mechanical fatigues tests due to the effective adhesion between the metal interlayer and amorphous WO_3−x_ layer and good interface between the Ag and WO_3−x_. The temperature profile of the WAW-based TFHs was compared with ITO based TFHs to substitute typical ITO-based TFHs. The WAW-based TFHs reaches high saturation temperatures (120 °C) at half the voltage (4 V) compared to ITO-based TFHs due to the lower sheet resistance. Moreover, we showed that WAW based TFHs are effective and have safer convection TFHs because of the higher convective heat transfer coefficient. Consequently, thermally evaporated optimized WAW multilayer is a promising candidate as a replacement for conventional TCEs, which were used to fabricate high-efficiency TFHs and optoelectronic devices due to the advantages of WAW multilayer like high transmittance, low sheet resistance, flexibility and high convective heat transfer coefficient.

## Methods

### Thermal evaporation of WO_3−x_, Ag, WO_3−x_/Ag/WO_3−x_ multilayer film

Before fabricating the WAW multilayer, we optimized the deposition rate of each layer in the WAW multilayer based on analysis of each WO_3−x_ and Ag single layer film according to the deposition rate, respectively. We fabricated the thermal evaporated WO_3−x_, Ag film with deposition rates from 0.25 nm/s to 1.0 nm/s with 0.25 nm/s interval as a variable. In the evaporation process, we used the thermal evaporation system (15NNS005, NNS Vacuum) at room temperature, and the base pressure of the vacuum chamber was under 1 × 10^−6^ Torr. The thickness of the WO_3−x_ and Ag layers were fixed at 30 nm and 12 nm, respectively, which is the same with the thickness of each layer of the WAW multilayer structure and controlled using a thickness monitor (STM-100/MF, Sycon) attached in the thermal evaporation system. The evaporation process of the WO_3−x_ film, deposition rate of 0.25 nm/s, 0.5 nm/s, 0.75 nm/s and 1.0 nm/s, respectively, by using a WO_3_ granular source (99.99 wt%). Other evaporation conditions, such as the Z-factor (0.529), tool factor (151%) and substrate rotation speed (11 rpm) were maintained to be identical in every deposition of WO_3−x_. In the evaporation process of the Ag film, the deposition rate was 0.25 nm/s, 0.5 nm/s, 0.75 nm/s and 1.0 nm/s, respectively, using Ag granular source (99.99 wt%). Other evaporation conditions, such as Z-factor (1.00), tool factor (159%) and substrate rotation speed (11 rpm) were maintained to be identical. The bottom WO_3−x_ layer, Ag interlayer and top WO_3−x_ layer were consecutively deposited on a CPI substrate to fabricate flexible and transparent conducting WAW multilayer film. The deposition process of each layer was using an identical evaporation system at room temperature without breaking the vacuum. We used the same evaporation conditions as the fabrication process of the WO_3−x_ and Ag single layer film. In the fabrication process, the bottom WO_3−x_ layer was evaporated with a fixed deposition rate of 0.25 nm/s onto the CPI substrate. The Ag interlayer with various deposition rates (0.25 nm/s, 0.5 nm/s, 0.75 nm/s and 1.0 nm/s) was evaporated onto the bottom WO_3−x_ layer. The top WO_3−x_ layer was deposited onto the Ag interlayer, and we used identical evaporation conditions for both the bottom and top WO_3−x_ layers to maximize the transmittance by forming a symmetric multilayer structure.

### Characterization of the WO_3−x_, Ag film and WO_3−x_/Ag/WO_3−x_ multilayer TCE

The electrical of the thermally evaporated WO_3−x_, Ag single layer films and WAW multilayer film were examined by using Hall measurements (HMS-4000AM, Ecopia). The optical properties were analyzed by using a UV/visible spectrometer (V-670, Jasco). The effect of the deposition rate on the surface morphology and cross-sectional analysis were investigated by using field emission scanning electron microscopy (JSM-7600F, Jeol) analysis. The depth-profile analysis of the WAW multilayer was using the X-ray photoelectron spectroscopy (K-alpha, Thermo U. K.). The work function of the optimized WAW multilayers film was analyzed by Kelvin probe force microscopy (XE-100, Park systems). The mechanical properties of the WAW multilayers film were analyzed by using a lab-designed bending test system. The outer and inner bending test was conducted to examine tensile and compressive stress durability on the film, respectively. In addition, the fatigue tests were performed by using a lab-designed bending, rolling and twisting test machine, for 10,000 cycles to demonstrate the durability of WAW multilayers film.

### Fabrication and assessments of the TFHs

In order to demonstrate the WAW multilayer is suitable as a transparent conducting electrode for TFHs, we applied an Ag paste at both edge sides of the TCE and laminate Cu tape on to that to use as contact electrode of the WAW TFHs, after fabrication of the thermally evaporated WAW multilayer on the CPI substrate (size of 25 × 25 mm^2^). In order to supply DC power to the WAW multilayer based TFHs, power supply (OPS 3010, ODA technologies) were used and applied voltage through the Cu contact electrode at the TFH edge side. The heating properties of the TFHs was measured by using a thermocouple and an IR thermal image camera (A35sc, FLIR). Moreover, to assess the efficiency of the WAW based TFHs, we calculated radiatively transferred heat and measured the convective heat transfer coefficient.

### Manuscript comments

One of the co-author (Sang-Hwi Lim) drew drawings in Fig. 1a,b, inset of Fig. 6a and inset of Fig. 7a using a **RHINO 6**, which is 6^th^ version of this drawing program. The inset of Fig. 2, and Fig. 4a,b,c,d and Fig. 7c are drawn by using a **Powerpoint** program. The URL link of the **RHINO** 6 drawing program and **Powerpoint** program is http://www.rhino3d.com and http://www.microsoft.com. In addition, we checked the terms of use of the software **RHINO 6** and **Powerpoint**. Photographs in Fig. 1 (c), 6 (b, c), 7 (b), 9 (c) were taken by one of the co-author (Sang-Hwi Lim)

## Supplementary information

Supplementary Information.

## References

[1] Cao W, Li J, Chen H, Xue J (2014). Transparent electrodes for organic optoelectronic devices: a review. J. Photonics Energy.

[2] Hecht DS, Hu L, Irvin G (2011). Emerging transparent electrodes based on thin films of carbon nanotubes, graphene, and metallic nanostructures. Adv. Mater..

[3] Cui Y, Hu LB, Kim HS, Lee JY, Peumans P (2010). Scalable Coating and Properties of Transparent, Flexible, Silver Nanowire Electrodes. ACS Nano.

[4] Lewis J, Grego S, Chalamala B, Vick E, Temple D (2004). Highly flexible transparent electrodes for organic light-emitting diode-based displays. Appl. Phys. Lett..

[5] Choo DC, Bae SK, Kim TW (2019). Flexible, transparent patterned electrodes based on graphene oxide/silver nanowire nanocomposites fabricated utilizing an accelerated ultraviolet/ozone process to control silver nanowire degradation. Sci. Rep..

[6] Li J (2014). A flexible and transparent thin film heater based on a silver nanowire/heat-resistant polymer composite. Macromol. Mater. Eng..

[7] Sui D (2011). Flexible and transparent electrothermal film heaters based on graphene materials. Small..

[8] Cheong HG, Kim JH, Song JH, Jeong U, Park JW (2015). Highly flexible transparent thin film heaters based on silver nanowires and aluminum zinc oxides. Thin Solid Films..

[9] Celle C (2012). Highly flexible transparent film heaters based on random networks of silver nanowires. Nano Res..

[10] Park SH (2016). Roll-to-Roll sputtered ITO/Cu/ITO multilayer electrode for flexible, transparent thin film heaters and electrochromic applications. Sci. Rep..

[11] Bel Hadj Tahar R, Ban T, Ohya Y, Takahashi Y (1998). Tin doped indium oxide thin films: Electrical properties. J. Appl. Phys..

[12] Kato K, Kuwahara M, Kawashima H, Tsuruoka T, Tsuda H (2017). Current-driven phase-change optical gate switch using indium-tin-oxide heater. Appl. Phys. Express.

[13] López-Naranjo EJ, González-Ortiz LJ, Apátiga LM, Rivera-Muñoz EM, Manzano-Ramírez A (2016). Transparent Electrodes: A Review of the Use of Carbon-Based Nanomaterials. J. Nanomater..

[14] Seok HJ, Lee JH, Park JH, Lim SH, Kim HK (2019). Transparent Conducting Electrodes for Quantum Dots Light Emitting Diodes. Isr. J. Chem..

[15] Na S-I (2008). Efficient and flexible ITO-free organic solar cells using highly conductive polymer anodes. Adv. Mater..

[16] Gupta D, Wienk MM, Janssen RAJ (2013). Efficient polymer solar cells on opaque substrates with a laminated PEDOT:PSS top electrode. Adv. Energy Mater.

[17] Jeong JA, Kim HK (2009). Low resistance and highly transparent ITO-Ag-ITO multilayer electrode using surface plasmon resonance of Ag layer for bulk-heterojunction organic solar cells. Sol. Energy Mater. Sol. Cells..

[18] Choi KH (2008). Highly flexible and transparent InZnSnO_x_/Ag/InZnSnO_x_ multilayer electrode for flexible organic light emitting diodes. Appl. Phys. Lett..

[19] Sahu DR, Lin SY, Huang JL (2006). ZnO/Ag/ZnO multilayer films for the application of a very low resistance transparent electrode. Appl. Surf. Sci..

[20] Jeong JA, Park YS, Kim HK (2010). Comparison of electrical, optical, structural, and interface properties of IZO-Ag-IZO and IZO-Au-IZO multilayer electrodes for organic photovoltaics. J. Appl. Phys..

[21] Cho D-Y (2015). Roll-to-roll sputtered Si-doped In_2_O_3_/Ag/Si-doped In_2_O_3_ multilayer as flexible and transparent anodes for flexible organic solar cells. J. Vac. Sci. Technol. A: Vacuum, Surfaces, and Film..

[22] Lee JE, Kim HK (2018). Highly transparent and flexible TiN doped In_2_O_3_ (ITON)/Ag-Ti/ITON multilayer electrodes coated on polyethylene terephthalate substrate. Thin Solid Films.

[23] Lee SM, Koo HW, Kim TW, Kim HK (2018). Asymmetric ITO/Ag/ZTO and ZTO/Ag/ITO anodes prepared by roll-to-roll sputtering for flexible organic light-emitting diodes. Surf. Coatings Technol..

[24] Seo HJ, Nah YC, Kim HK (2018). Roll-to-roll sputtered and patterned Cu_2-x_O/Cu/Cu_2-x_O multilayer grid electrode for flexible smart windows. RSC Adv..

[25] Yin Y, Lan C, Guo H, Li C (2016). Reactive Sputter Deposition of WO_3_/Ag/WO_3_ Film for Indium Tin Oxide (ITO)-Free Electrochromic Devices. ACS Appl. Mater. Interfaces.

[26] Kim DH, Cho KS, Kim HK (2017). Thermally evaporated indium-free, transparent, flexible SnO_2_/AgPdCu/SnO_2_ electrodes for flexible and transparent thin film heaters. Sci. Rep..

[27] Cho KS, Kim HK (2018). Transparent and flexible Sb-doped SnO_2_ films with a nanoscale AgTi alloyed interlayer for heat generation and shielding applications. RSC Adv..

[28] Cattin L (2010). Investigation of low resistance transparent MoO_3_/Ag/MoO_3_ multilayer and application as anode in organic solar cells. Thin Solid Films.

[29] Kim S, Lee J-L (2012). Design of dielectric/metal/dielectric transparent electrodes for flexible electronics. J. Photonics Energy.

[30] Liu X, Cai X, Mao J, Jin C (2001). ZnS/Ag/ZnS nano-multilayer films for transparent electrodes in flat display application. Appl. Surf. Sci..

[31] Seok HJ, Jang HW, Lee DY, Son BG, Kim HK (2019). Roll-to-roll sputtered, indium-free ZnSnO/AgPdCu/ZnSnO multi-stacked electrodes for high performance flexible thin-film heaters and heat-shielding films. J. Alloys Compd..

[32] Liu SW (2016). ITO-free, efficient, and inverted phosphorescent organic light-emitting diodes using a WO_3_/Ag/WO_3_ multilayer electrode. Org. Electron..

[33] Liang F (2018). Promising ITO-free perovskite solar cells with WO_3_-Ag-SnO_2_ as transparent conductive oxide. J. Mater. Chem. A.

[34] Cao W (2012). Flexible organic solar cells using an oxide/metal/oxide trilayer as transparent electrode. Org. Electron. physics, Mater. Appl..

[35] Meyer J (2012). Transition Metal Oxides for Organic Electronics: Energetics, Device Physics and Applications. Adv. Mater..

[36] Hung LS, Liao LS, Lee CS, Lee ST (1999). Sputter deposition of cathodes in organic light emitting diodes. J. Appl. Phys..

[37] Kim HK (2004). Plasma damage-free deposition of Al cathode on organic light-emitting devices by using mirror shape target sputtering. Appl. Phys. Lett..

[38] Kim HK (2005). Plasma damage-free sputtering of indium tin oxide cathode layers for top-emitting organic light-emitting diodes. Appl. Phys. Lett..

[39] Hong K (2011). Optical properties of WO_3_/Ag/WO_3_ multilayer as transparent cathode in top-emitting organic light emitting diodes. J. Phys. Chem. C.

[40] Zhang N, Hu Y, Liu X (2013). Transparent organic thin film transistors with WO_3_/Ag/WO_3_ source-drain electrodes fabricated by thermal evaporation. Appl. Phys. Lett..

[41] Yang HU (2015). Optical dielectric function of silver. Phys. Rev. B - Condens. Matter Mater. Phys..

[42] Haacke G (1976). New figure of merit for transparent conductors. J. Appl. Phys..

[43] Lee HK, Na JY, Moon YJ, Seong TY, Kim SK (2015). Design of near-unity transmittance dielectric/Ag/ITO electrodes for GaN-based light-emitting diodes. Curr. Appl. Phys..

[44] Yang S (2017). Transparent WO_3_/Ag/WO_3_ electrode for flexible organic solar cells. Mater. Lett..

[45] Yoon KH, Lee JW, Cho YS, Kang DH (1995). Structural and photocurrent-voltage characteristics of tungsten oxide thin films on p-GaAs. Appl. Phys. Lett..

[46] Han S (2009). Improving performance of organic solar cells using amorphous tungsten oxides as an interfacial buffer layer on transparent anodes. Org. Electron..

[47] Hutchins MG, Abu-Alkhair O, El-Nahass MM, Abdel-Hady K (2006). Electrical conduction mechanisms in thermally evaporated tungsten trioxide (WO_3_) thin films. J. Phys. Condens. Matter.

[48] Indluru A, Alford TL (2009). Effect of Ag thickness on electrical transport and optical properties of indium tin oxide-Ag-indium tin oxide multilayers. J. Appl. Phys..

[49] Ye, M. *et al*. Recent advances in interfacial engineering of perovskite solar cells. *J. Phys. D. Appl. Phys*. **50** (2017).

[50] Liu D, Kelly TL (2014). Perovskite solar cells with a planar heterojunction structure prepared using room-temperature solution processing techniques. Nat. Photonics.

[51] Correa Baena JP (2015). Highly efficient planar perovskite solar cells through band alignment engineering. Energy Environ. Sci..

[52] Khyzhun OY (2000). XPS, XES and XAS studies of the electronic structure of tungsten oxides. J. Alloys Compd..

[53] Hüfner S, Wertheim GK, Smith NV, Traum MM (1972). XPS density of states of copper, silver, and nickel. Solid State Commun..

[54] Li H, Lv Y, Zhang X, Wang X, Liu X (2015). High-performance ITO-free electrochromic films based on bi-functional stacked WO_3_/Ag/WO_3_ structures. Sol. Energy Mater. Sol. Cells.

[55] Bae JJ (2012). Heat dissipation of transparent graphene defoggers. Adv. Funct. Mater..

[56] Koubli E, Tsakanikas S, Leftheriotis G, Syrrokostas G, Yianoulis P (2015). Optical properties and stability of near-optimum WO_3_/Ag/WO_3_ multilayers for electrochromic applications. Solid State Ionics.

[57] Sun K (2011). Effect of the heat treatment on the infrared emissivity of indium tin oxide (ITO) films. Appl. Surf. Sci..

