# Supplementary material for: Deposition Rate Effect on Optical and Electrical Properties of Thermally Evaporated WO3−x/Ag/WO3−x Multilayer Electrode for Transparent and Flexible Thin Film Heaters
Source: Sci Rep. 2020 May 20;10:8357. doi: 10.1038/s41598-020-65260-1 (PMC7239933; doi:10.1038/s41598-020-65260-1)
Supplement: Supplementary file 1 — Supplementary Information. [file 41598_2020_65260_MOESM1_ESM.docx]

**Supporting Information**

Deposition Rate Effect on Electrical and Optical Properties of Thermally Evaporated WO_3-x_/Ag/WO_3-x_ Multilayer Electrode for Flexible and Transparent Thin Film Heaters

**Sang-Hwi Lim and Han-Ki Kim***

^a^School of Advanced Materials Science and Engineering, Sungkyunkwan University, 2066, Seobu-ro, Jangan-gu, Suwon-si, Gyeonggi-do, 16419, Republic of Korea

[***^*^hankikim@skku.***](mailto:*hankikim@skku.)***edu***


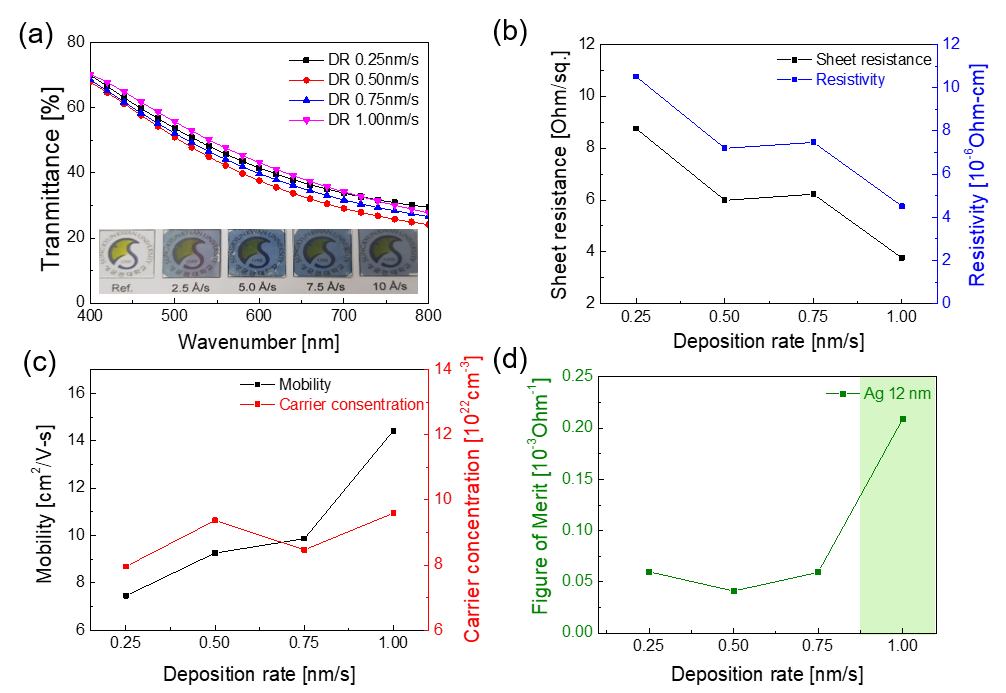


**Figure S1.** (a) Transmittance, (b) sheet resistance, resistivity, (c) carrier mobility, and carrier concentration of thermally evaporated Ag single layer with increasing deposition rate from 0.25 to 1.0 nm/sec. (d) Figure of merit (FOM=T^10^/R_sh_) values calculated from sheet resistance (R_sh_) and optical transmittance (T) of the evaporated Ag films.


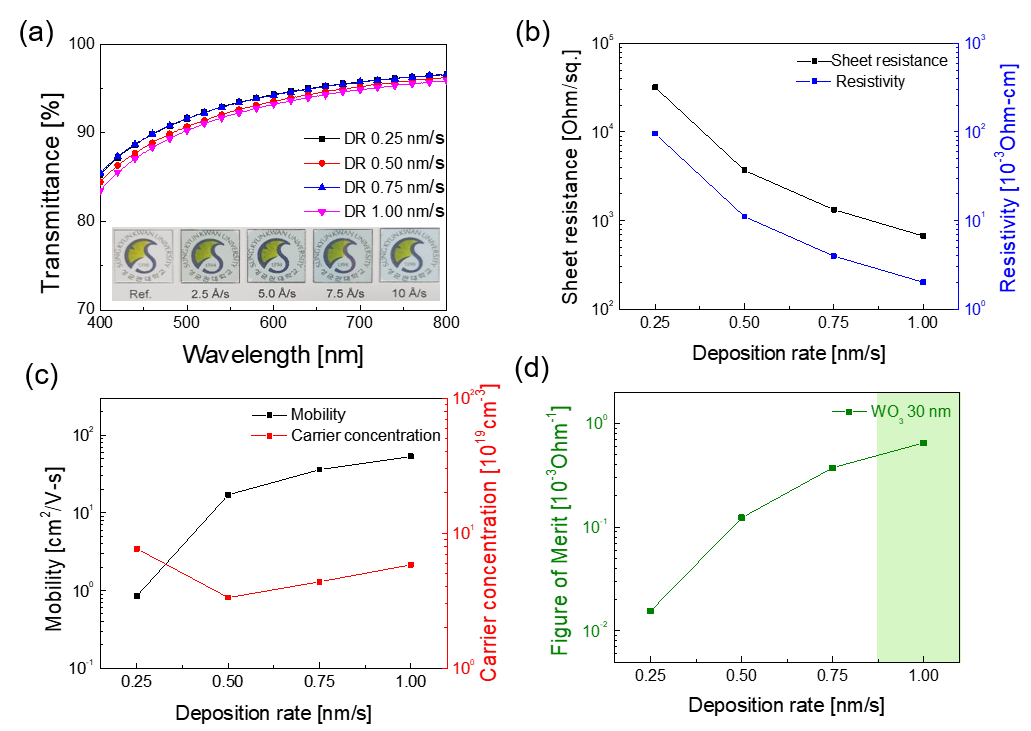
**Figure S2.** (a) Transmittance, (b) Sheet resistance and resistivity and (c) mobility and carrier concentration of thermally evaporated WO_3-x_ single layer on a glass substrate with increasing deposition rate from 0.25 nm/s to 1.0 nm/s. (d) FOM values calculated from sheet resistance (R_sh_) and optical transmittance (T) of the WO_3-x_ films.


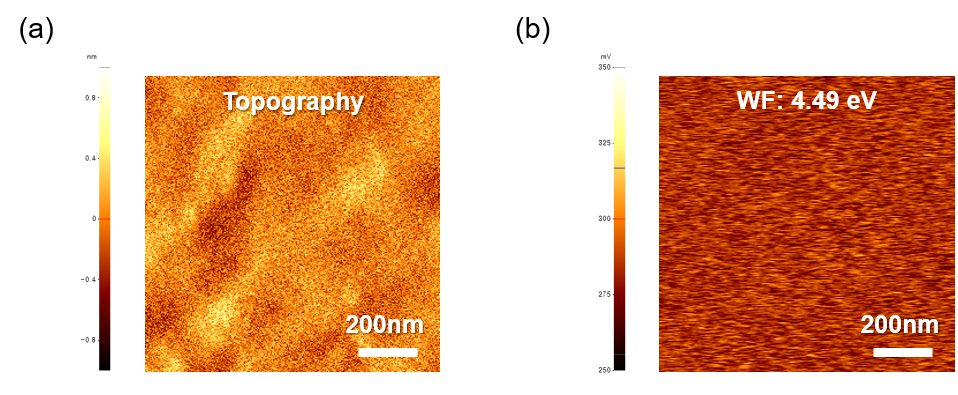
**Figure S3.** (a) Topography and (b) Work function of optimized WAW multilayer film on a glass substrate were measured using a Kelvin probe force microscopy.

**
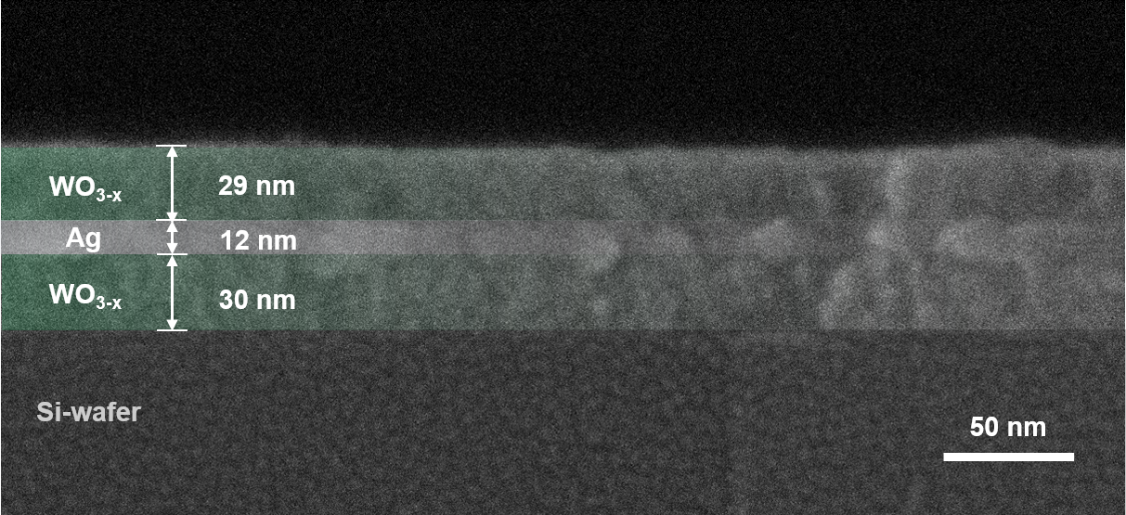
**

**Figure S4.** Cross sectional FE-SEM image of optimized WAW multilayer film on a Si-wafer.

**Table S1**. Materials, sheet resistance, saturation temperature with applied voltage and emissivity coefficients of our WAW, ITO and reported TCE based TFHs.

| TCE materials | Sheet resistance (Ohm/  square) | Saturation temperature (°C) | Volt-age  (V) | Convective heat transfer coefficient [W/cm^2^C] | Notes | Ref. |
| --- | --- | --- | --- | --- | --- | --- |
| WO_3-x_/Ag/  WO_3-x_ | 3.77 | 120 | 4 | 55^*^ | Evaporation | This work, 1^*^ |
| ITO | 45.3 | 117 | 8 | 35^*^ | Sputtering | This work, 2^*^ |
| AgNWs/  polymer | 25 | 110 | 7 | - | Drop cast | 3 |
| AgNWs/  AZO | 30.0 | 130 | 6 | - | Bar coating/  Sputtering | 4 |
| SWCNT | 22600 | 47.4 | 60 | 25 | Dip coating | 5 |
| Graphene film | 641 | 206 | 60 | 12.4^*^ | Spin coating | 6, 7^*^ |
| ITO/Cu/ITO | 11.8 | 110.2 | 8.5 | - | R2R sputter | 8 |
| ATO/Ag-Ti/  ATO | 6.91 | 102.8 | 4.6 | - | Evapoartion | 9 |
| Cr thin film | - | - | - | 13 | E-beam evaporation | 7 |
| Pt thin film |  |  |  | 36 | Sputtering | 5 |

References

1. Koubli, E., Tsakanikas, S., Leftheriotis, G., Syrrokostas, G. & Yianoulis, P. Optical properties and stability of near-optimum WO_3_/Ag/WO_3_ multilayers for electrochromic applications. *Solid State Ionics* **272**, 30–38 (2015).

2. Sun, K. *et al.* Effect of the heat treatment on the infrared emissivity of indium tin oxide (ITO) films. *Appl. Surf. Sci.* **257**, 9639–9642 (2011).

3. J. Li, J. Liang, X. Jian, W. Hu, J. Li, Q. Pei, A flexible and transparent thin film heater based on a silver nanowire/heat-resistant polymer composite. *Macromol. Mater. Eng*. **299**, 1403–1409 (2014).

4. H.G. Cheong, J.H. Kim, J.H. Song, U. Jeong, J.W. Park, Highly flexible transparent thin film heaters based on silver nanowires and aluminum zinc oxides. *Thin Solid Films*. **589**, 633–641 (2015).

5. Kang, T. J., Kim, T., Seo, S. M., Park, Y. J. & Kim, Y. H. Thickness-dependent thermal resistance of a transparent glass heater with a single-walled carbon nanotube coating. *Carbon N. Y.* **49**, 1087–1093 (2011).

6. D. Sui, Y. Huang, L. Huang, J. Liang, Y. Ma, Y. Chen, Flexible and transparent electrothermal film heaters based on graphene materials. *Small*. **7**, 3186–3192 (2011).

7. Bae, J. J. *et al.* Heat dissipation of transparent graphene defoggers. *Adv. Funct. Mater.* **22**, 4819–4826 (2012).

8. S.H. Park, S.M. Lee, E.H. Ko, T.H. Kim, Y.C. Nah, S.J. Lee, J.H. Lee, H.K. Kim, Roll-to-Roll sputtered ITO/Cu/ITO multilayer electrode for flexible, transparent thin film heaters and electrochromic applications. *Sci. Rep*. **6** 1–12 (2016).

9. Cho, K. S. & Kim, H. K. Transparent and flexible Sb-doped SnO_2_ films with a nanoscale AgTi alloyed interlayer for heat generation and shielding applications. *RSC Adv.* **8**, 2599–2609 (2018).
